# Serosorting and HIV/STI Infection among HIV-Negative MSM and Transgender People: A Systematic Review and Meta-Analysis to Inform WHO Guidelines

**DOI:** 10.1155/2013/583627

**Published:** 2013-04-14

**Authors:** Caitlin E. Kennedy, Laura J. Bernard, Kathryn E. Muessig, Kelika A. Konda, Elie A. Akl, Ying-Ru Lo, Antonio Gerbase, Kevin R. O'Reilly

**Affiliations:** ^1^Department of International Health, Johns Hopkins Bloomberg School of Public Health, Baltimore, MD 21205, USA; ^2^Social and Behavioral Interventions Program, Department of International Health, Room E5033, Johns Hopkins Bloomberg School of Public Health, 615 North Wolfe Street, Baltimore, MD 21205, USA; ^3^Department of Medicine, University of North Carolina, Chapel Hill, NC 27599, USA; ^4^Instituto de Estudios en Salud, Sexualidad y Desarrollo Humano, Universidad Peruana Cayetano Heredia, Lima, Peru; ^5^Department of Medicine, State University of New York at Buffalo, Buffalo, NY 14214, USA; ^6^Department of Clinical Epidemiology and Biostatistics, McMaster University, Hamilton, ON L8S 4L8, Canada; ^7^Department of HIV/AIDS, World Health Organization, 1211 Geneva, Switzerland

## Abstract

We conducted a systematic review and meta-analysis to assess the association between serosorting and HIV infection, sexually transmitted infections (STIs), and quality of life among men who have sex with men (MSM) and transgender people. Two reviewers independently screened abstracts and abstracted data. Meta-analyses were conducted using random effects models. Of 310 citations reviewed, 4 observational studies, all with MSM, met inclusion criteria. Compared to consistent condom use, serosorting was associated with increased risk of HIV (3 studies, odds ratio (OR): 1.80, 95% confidence interval (CI):1.21–2.70) and bacterial STIs (1 study, OR: 1.62, 95% CI: 1.44–1.83). Compared to no condom use, serosorting was associated with reduced risk of HIV (3 studies, OR: 0.46, 95% CI: 0.25–0.83) and bacterial STIs (1 study, OR: 0.81, 95% CI: 0.73–0.91). Among HIV-negative MSM, condom use appears to be more protective against HIV and STIs than serosorting and should be encouraged. However, serosorting may be better than no condom use as a harm reduction strategy.

## 1. Introduction

Serosorting occurs when individuals limit their sexual partners to those with the same HIV status as themselves [1]. Generally, individuals seek out seroconcordant partners in order to forgo condom use while still reducing the risk of HIV [2–4]. This is known as partner serosorting or pure serosorting, while condom serosorting refers to the selective use of condoms with HIV serodiscordant partners [4]. Serosorting is one of a variety of seroadaptive behaviors, also called sexual harm reduction strategies. Seroadaptive behaviors occur when individuals alter their sexual behaviors based on knowledge or suspected knowledge of HIV serostatus of their partners to reduce, but not eliminate, the risk of HIV transmission or acquisition within a known or suspected serodiscordant couple. Seroadaptive behaviors include serosorting, seropositioning, withdrawal before ejaculation, and negotiation around viral load and may include other behaviors not yet identified.

Serosorting and seroadaptive behaviors have received wider attention in recent years as expansion in HIV testing and treatment worldwide has meant that more people living with HIV know their status, are living longer and healthier lives, and resume or continue sexual activity. At the same time, there has been continued debate on the protective effect of serosorting on HIV and sexually transmitted infection (STI) acquisition among HIV-negative individuals. While serosorting may contribute to reducing transmission of HIV, modeling suggests that incomplete knowledge or disclosure of serostatus—among other factors—may limit its effectiveness as a prevention strategy [5, 6].

Others warn that serosorting may increase HIV-positive individuals' risk for acquiring additional strains of HIV (superinfection) [10], which in turn could increase incidence of HIV coinfection (becoming infected with multiple strains of HIV at initial infection) [11]. Both superinfection and coinfection raise concerns about increased virulence, drug resistance, and treatment failure [10, 11]. Another caution is the inability of serosorting to prevent transmission of other sexually transmitted infections [12].

In 2009, the World Health Organization (WHO) began a process to develop guidelines for the prevention and treatment of HIV and other STIs among men who have sex with men (MSM) and transgender people [13]. In preparation for these guidelines, we conducted a systematic review and meta-analysis of studies reporting the association between serosorting and HIV infection, STI infection, or quality of life among HIV-negative MSM or transgender people.

## 2. Materials and Methods

Following established guidelines [14], we conducted and report on a comprehensive systematic review of articles on serosorting and seroadaptive behaviors. This larger review covered a wide variety of publications, including descriptive epidemiological studies, studies of interventions promoting serosorting, modeling exercises, qualitative studies, review articles, and think pieces. Full protocol details are available in the WHO guidelines annexes [13]. We focus here on the articles that examined the relationship between serosorting and HIV infection, STI infection, or quality of life among HIV-negative MSM or transgender people.

### 2.1. Inclusion Criteria

To be included in this analysis, articles had to meet each of the following criteria:

1. published in a peer-reviewed journal,
2. population: HIV-negative MSM or transgender people,
3. intervention: serosorting,
4. comparison: condom use, or no condom use,
5. outcomes of interest: HIV infection, STI infection, and quality of life,
6. study design: case-control or cohort studies reporting a measure of association between serosorting and at least one of the outcomes of interest.

We used no restrictions based on location of studies or language of publication. 

### 2.2. Search Strategy and Selection Criteria

We searched the following five electronic databases from January 1990 to April 2010: PubMed, PsycINFO, Sociological Abstracts, CINAHL (Cumulative Index to Nursing and Allied Health Literature), and EMBASE. We used the following search terms: serosort∗; seroposition∗; seroadapt∗; sero-sort∗; sero-position∗; sero-adapt∗; HIV and “partner selection”; AIDS and “partner selection”; HIV and “partner choice”; AIDS and “partner choice”; “strategic positioning”; “sexual harm reduction”; and seroguessing.

To identify additional articles that might have been missed through electronic database searching we hand-searched the tables of contents of the following five journals: AIDS, JAIDS, AIDS and Behavior, AIDS Education and Prevention, and AIDS Care. We also screened the references of all articles included in the review. Finally, we contacted selected experts in the field—in particular, members of the WHO guideline development group—to identify additional references. 

We downloaded citations into bibliographic management software (EndNote V.10) and utilized a three-step screening process. First, one reviewer screened the titles and abstracts of all citations to exclude those that clearly did not meet the inclusion criteria. Second, two reviewers screened in duplicate and independently the remaining citations, compared their results, and resolved discrepancies through discussion and full text review. Third, the full text of included articles was read to ensure eligibility and correct study classification.

### 2.3. Data Extraction

Each article was read, and data were extracted by two members of the study team working independently. They resolved discrepancies through discussion and consensus. The following data were systematically extracted from each study: study identification information; serosorting definition; years of study; location, setting, and target group; age range and gender; sample size; sampling strategy; study design; length of followup; outcome measures; comparison groups; effect sizes; confidence intervals; significance levels; funding source; and limitations. We assessed study methodological quality using a series of items developed for other HIV behavioral intervention systematic reviews [15, 16] and the GRADE system for rating the quality of evidence for each outcome. GRADE uses four categories to rate the quality of evidence from high to very low; randomized controlled trials start as high-quality evidence and observational studies as low-quality evidence, while different factors may increase or decrease the final rating [17].

### 2.4. Meta-Analysis

We followed standard meta-analytic methods to derive standardized effect size estimates [18] and used the software package Comprehensive Meta-Analysis, Version 2.2 to conduct statistical analyses. For studies that compared two groups and reported dichotomous outcomes, we first converted effect size estimates to the common metric of an odds ratio. Odds ratios were pooled using random effects models. Only one study included in the meta-analysis conducted analyses adjusted for confounders, but these adjusted analyses were presented for only one comparison of interest. For comparability across analyses and across studies, we used unadjusted data in all meta-analyses but present results using adjusted data as well. In one case, data were presented as clinic visits rather than individual participants. We assessed heterogeneity using the *I*-squared statistic with a significance level set at *P* = 0.05. 

## 3. Results

### 3.1. Study Descriptions

**Figure 1:**
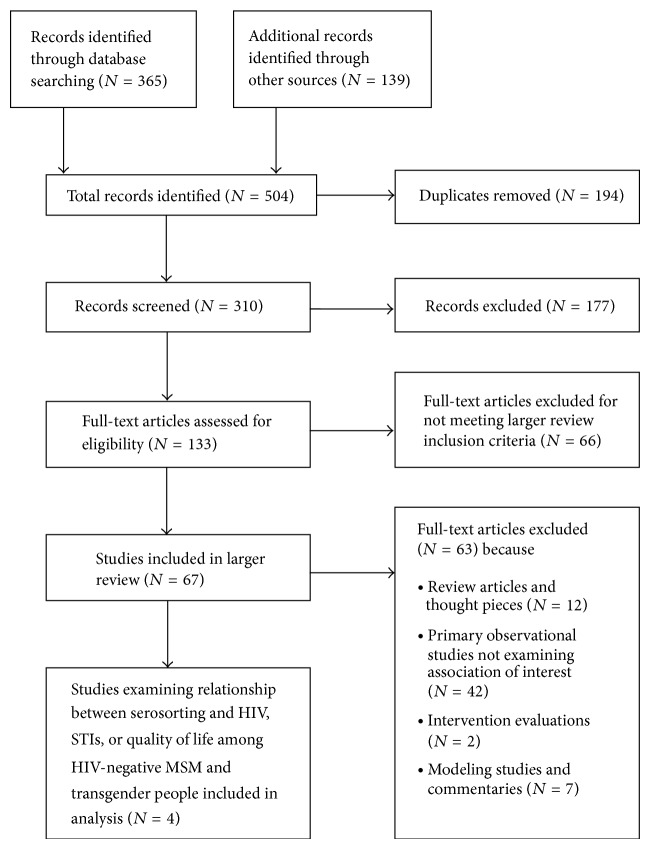
Disposition of articles in the search and screening process.

Of 310 citations reviewed, four observational studies met the inclusion criteria (Figure 1) [3, 7–9]. All four studies were conducted in urban settings in high-income countries: three in the United States and one in Australia (Table 1). All were conducted among MSM; none of the studies explicitly included or excluded transgender people.

### 3.2. Serosorting Measures

All four studies defined serosorting by using reported behavior, regardless of whether this behavior was intentional or unintentional. Three studies [3, 7, 8] defined serosorting among HIV-negative men as unprotected anal intercourse (UAI) with HIV-negative partners only (partner serosorting). These studies compared serosorting to consistent condom use and no condom use. For one study [3], condom use data were extracted from the category “no unprotected anal intercourse,” which included both consistent condom users and nonsexually active individuals. We contacted the authors of this study who provided us with revised data for just consistent (100%) condom users, which were used in meta-analysis and thus yield slightly different effect estimates than reported in the original article.

The fourth study [9] measured serosorting only among HIV-negative MSM who reported both seroconcordant and serodiscordant partners. The authors calculated a serosorting score as the odds of condom use with HIV-positive partners or partners of unknown HIV status, divided by the odds of condom use with HIV-negative partners (condom serosorting). Because this study defined serosorting differently than the other three studies, we did not include it in the meta-analyses and present its results separately.

### 3.3. Study Rigor

All studies were observational, longitudinal cohort studies; due to the nature of serosorting, there were no randomized trials. Two studies controlled for potential confounders by adjusting analyses for differences between serosorters and nonserosorters (Table 1) [8, 9]. In the GRADE system, all outcomes were rated as “very low” quality due to factors described below.

### 3.4. Outcomes

#### 3.4.1. HIV Infection

**Figure 2:**
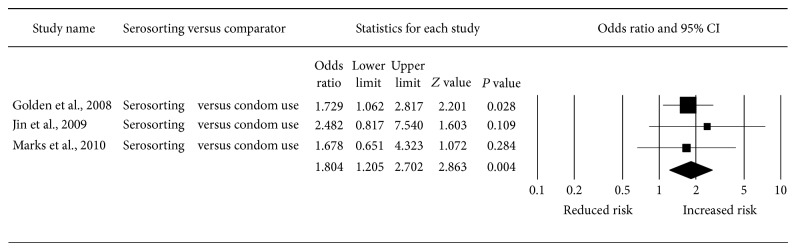
Meta-analysis of odds of HIV infection associated with serosorting versus condom use.

**Figure 3:**
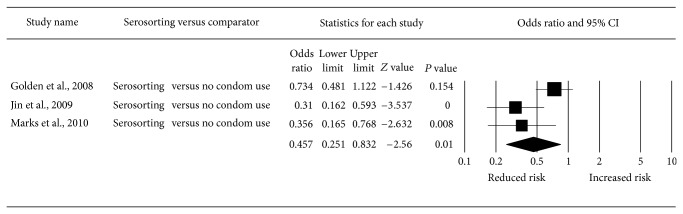
Meta-analysis of odds of HIV infection associated with serosorting versus no condom use.

All four studies measured the association between serosorting and HIV infection. Meta-analysis of three studies [3, 7, 8] found that, compared to consistent condom use, serosorting was associated with an increased risk of HIV (odds ratio (OR): 1.80, 95% confidence interval (CI): 1.21–2.70) (Figure 2). Heterogeneity of results was not significant (*I*-squared: 0.00%, *P* = 0.83). Compared to no condom use, serosorting was associated with a reduced risk of HIV (OR: 0.46, 95% CI: 0.25–0.83) (Figure 3). Heterogeneity of results was borderline significant (*I*-squared: 66.23%, *P* = 0.05). Using adjusted data from one study for serosorting compared to no condom use would have made almost no difference in the meta-analytic result (OR: 0.47, 95% CI: 0.26–0.85). 

The fourth study [9] found that serosorting was associated with a 12% decreased risk of HIV seroconversion for each one unit increase in the natural log serosorting score (OR: 0.88; 95% CI: 0.81–0.95), where a higher serosorting score indicated a greater likelihood of using condoms with HIV-positive or unknown partners as compared with HIV-negative partners. There was no difference in the protective effect of serosorting when analyses were stratified by the reported number of sexual partners.

According to the GRADE system, the quality of evidence for the association between serosorting and HIV infection was rated very low. Although there was no serious imprecision or inconsistency, the quality was rated down for the possibility of significant uncontrolled confounding by number and types of sex partners and for indirectness, because all studies were conducted in high-income settings where HIV testing is widely available, while serosorting relies on frequency and availability of high-quality HIV testing services, and because other important differences across settings such as stigma and HIV treatment rates could affect the association between serosorting and HIV infection.

#### 3.4.2. STI Infection

One study examined the association between serosorting and STI infection, measured as urethral or rectal gonorrhea or Chlamydia infection or early syphilis [7]. In this study, compared to consistent condom use, serosorting was associated with an increased risk of bacterial STIs (OR: 1.62, 95% CI: 1.44–1.83). Compared to no condom use, serosorting was associated with a reduced risk of bacterial STIs (OR: 0.81, 95% CI: 0.73–0.91). In GRADE, the quality of evidence for this outcome was rated very low for the same reasons as listed above.

#### 3.4.3. Quality of Life

No studies examined the association between serosorting and quality of life outcomes.

## 4. Discussion

Results from this systematic review and meta-analysis suggest that, among HIV-negative MSM, condom use is more protective against HIV and STIs than serosorting. However, as a harm reduction strategy, serosorting may be better than no condom use. These results are based on a small number of observational studies that measured serosorting behavior, intentional or not, rather than deliberate serosorting, and the possibility of significant confounding remains. 

Based on these findings and a discussion about the balance of risks and benefits, community values and preferences, costs, and feasibility of serosorting during the consensus conference, WHO issued the following recommendations.

1. Using condoms consistently is strongly recommended over serosorting for HIV-negative MSM and transgender people (strong recommendation, very low-quality evidence).
2. Serosorting is suggested over not using condoms among HIV-negative MSM and transgender people under specific circumstances as a harm reduction strategy (conditional recommendation, very low-quality evidence).

These specific circumstances were judged as essential for the effectiveness of serosorting and include availability of quality-assured HIV testing, high frequency of repeat HIV testing, and the existence of legal and social environments supportive of HIV testing and serostatus disclosure. 

The conditionality of the second recommendation is due to the low quality of available evidence and the indirectness of the studied samples to broader populations of MSM and transgender people. Existing evidence comes from populations in high-income countries where the qualifying circumstances are more likely to apply. The effectiveness of serosorting against HIV transmission is contingent on accurate, current knowledge of HIV status and ability to disclose. Thus, serosorting may have a less-protective effect in low-income countries if quality-assured HIV testing is limited and the environment is less supportive of HIV testing and serostatus disclosure. Additionally, serosorting in settings of higher overall HIV prevalence and lower rates of care and treatment coverage (thus higher community viral load) is likely to be associated with higher risk of HIV acquisition. The effect of serosorting on STI infection is less clear as the quality of evidence was very low. Although one study found a protective effect, serosorting on HIV status alone does not provide a mechanism for protection against other STI. Caution is warranted, particularly in settings where routine screening for asymptomatic urethral and rectal STI is not available. 

Although serosorting showed benefit when compared to no condom use, there was harm shown when compared to consistent condom use. Serosorting may be a potential harm reduction strategy for individuals who choose not to use condoms, but it should not be promoted as an alternative strategy for effective HIV prevention. Condoms are a much more effective means of preventing HIV acquisition and should be used and promoted. There is a need for increased availability of high-quality condoms and high-quality HIV testing and counseling in all settings, especially in low- and middle-income countries. 

Individuals using serosorting as a harm reduction strategy should still be screened regularly for HIV and STIs. Moreover, counseling offered to MSM and transgender people should address facts about serosorting, explain its potential utility and potential harms, highlight the very low quality of supporting evidence, and clarify any misconceptions that might increase such harms among potential users. 

This study was limited in scope as it only examined serosorting among HIV-negative MSM and transgender people. We did not examine outcomes of serosorting among HIV-positive individuals or heterosexuals, and we did not examine outcomes of other seroadaptive behaviors, including seropositioning, withdrawal before ejaculation, and selective sex with HIV-infected partners with undetectable viral loads. The protective effects of serosorting in other populations and other seroadaptive behaviors are unknown. These evolutions in seroadaptive strategies among MSM could affect estimates for risk of seroconversion, and ongoing epidemiological monitoring of the effectiveness of these emerging strategies is warranted.

In meta-analysis, we assessed heterogeneity using the *I*-squared statistic. However, this approach has low power to detect heterogeneity in cases where there are few included studies, as was the case in this review, so some authors choose to use *P* = 0.10 as a cutoff instead [19]. Had we done this, the heterogeneity of the results of serosorting compared to no condom use for HIV infection would have become significant, although this would not have affected the overall conclusions of the review. 

The studies included in this review had several limitations. First, all included studies were observational cohort studies. Individuals who choose to serosort and those who do not are likely to be different in potentially important ways. Although studies adjusted for several potential confounders, the overall association between serosorting and HIV/STI acquisition may be related to a variety of measured and unmeasured factors. Second, the included studies were conducted in high-income countries among mostly gay-identified MSM; no studies were conducted in low- or middle-income countries, and none specifically included transgender people. Restrictive social, legal, and policy environments may limit the settings where studies on serosorting among MSM and transgender people can be conducted. Third, one study used clinic visits rather than participants as the denominator, although HIV is an individual-level outcome. It is unclear how this might have affected the association between serosorting and HIV. Fourth, quality of life was considered an important outcome by the WHO guidelines development group but was not measured in any of the included studies. Fifth, serosorting in these studies included mutually monogamous seronegative relationships; excluding these relationships would likely increase the odds of HIV transmission associated with serosorting. Sixth, all included studies defined serosorting by actual behavior, regardless of whether the serosorting was deliberate. These data, therefore, may not accurately reflect the risk associated with intentional serosorting. Finally, these studies do not attempt to capture the multiple and complex nature of partner selection, which includes factors such as emotional intimacy, trust, and attraction, amongst many others. Further, the experience and implications of serosorting are likely to vary across settings according to both local health care conditions and local understandings of HIV testing, serostatus disclosure, and the nature of MSM and transgender relationships.

Serosorting is a dynamic field of research. Since the work to develop WHO guidelines was conducted, at least one additional study has been published which would have met the inclusion criteria for this review [20]; findings from this new study support the conclusions of this meta-analysis. WHO guidelines are scheduled to be regularly updated, and future efforts should continue to synthesize the evolving evidence base.

This study has important implications for research. There is a need for additional high-quality prospective observational studies assessing the specific benefits and harms of different behaviors to inform counseling strategies and increase their usefulness as harm reduction strategies. Future studies could also compare different counseling strategies around serosorting and other harm reduction behaviors. There is also a need to develop and validate tools for the measurement of quality of life in relation to sexual behavior in different populations including MSM and transgender people.

## 5. Conclusions

Serosorting for HIV-negative MSM and transgender persons cannot be recommended over consistent condom use. However, under specific circumstances for individuals who choose not to or are unable to use condoms, serosorting may be preferable to nothing as a harm reduction strategy. 

## Figures and Tables

**Table 1:** Study descriptions and key findings.

Study	Population	Findings
Golden et al., 2008 [7]	HIV-negative MSM^1^ in Seattle, USA 8,314 participants	HIV incidence (among men who tested HIV negative in past year) (i) Condom use (only protected anal intercourse): 28/1827 clinic visits (1.5%) (ii) Serosorting (UAI^2^ with HIV negative only): 40/1526 clinic visits (2.6%) (iii) No condom use (nonconcordant UAI): 49/1386 clinic visits (3.5%)
	Age: HIV men: <24: 19% 25–29: 20% 30–34: 16% 35–39: 17% >40: 28%	STI incidence (Bacterial STI^3^: urethral or rectal gonorrhea or chlamydia or early syphilis) (i) Condom use: 601/3859 clinic visits (16%) (ii) Serosorting: 738/3201 clinic visits (23%) (iii) No condom use: 831/3088 clinic visits (27%)

Jin et al., 2009 [3]	HIV-negative MSM in Sydney, Australia	HIV incidence (median followup 3–9 years) (i) Condom use (only protected anal intercourse; data received from authors for only consistent condom users); serosorting (UAI with HIV negative only); no condom use (UAI with some unknown or any HIV positive)
	1427 participants Median age: 35 (range: 18–75)	(ii) Serosorting versus condom use: hazard ratio 2.56; 95% CI^4^ (0.84–7.78) (iii) Serosorting versus no condom use: hazard ratio 0.31; 95% CI (0.16–0.59)

Marks et al., 2010 [8]	HIV-negative black and Latino MSM in New York, LA, and Philadelphia, USA 724 participants Age not reported	HIV incidence (testing positive during study among those reporting a previous HIV-negative test, most within past year) (i) Condom use (only protected anal intercourse); serosorting (UAI with HIV negative only); No condom use (UAI not limited to HIV negative partners) (ii) Serosorting versus condom use: unadjusted odds ratio 1.68; 95% CI (0.65–4.32) (iii) No condom use versus serosorting: unadjusted odds ratio 2*·*81; 95% CI (1.30–6.05); adjusted∗ odds ratio 2.54; 95% CI (1.14–5.68) ^*^Adjusted for number of UAI partners of any serostatus, age, education, and employment status

Philip et al., 2010 [9]	HIV-negative MSM in six cities in the EXPLORE study, USA 4295 participants in EXPLORE study; 2623 participants used for serosorting analyses Age: <30: 42% 31–39: 39% >40: 20%	HIV incidence (i) One-unit increase in the natural log serosorting score (odds of condom use with HIV-positive/unknown partners divided by odds of condom use with HIV negative partners): odds ratio 0.88; 95% CI (0.81–0.95) ^*^Adjusted for race/ethnicity, numbers of sexual partners, self-report of sexually transmitted infections, methamphetamine and heavy alcohol use, depression, and use of drug and alcohol during sex

^1^MSM: men who have sex with men.

^2^UAI: unprotected anal intercourse.

^3^STI: sexually transmitted infection.

^4^CI: confidence interval.

## Key Messages

- To date, no work has reviewed the literature to examine whether serosorting based on HIV status is associated with improved outcomes.
- This study examines the association between serosorting and HIV infection, STIs, and quality of life among men who have sex with men and transgender people.
- Serosorting was associated with increased risk of HIV and bacterial STIs compared to consistent condom use, but reduced risk compared to no condom use.
- Condom use appears to be more protective against HIV and STIs than serosorting and should be encouraged. However, serosorting may be better than no condom use as a harm reduction strategy.

## References

[1] Eaton L. A., Kalichman S. C., Cain D. N. (2007). Serosorting sexual partners and risk for HIV among men who have sex with men. *The American Journal of Preventive Medicine*.

[2] Elford J. (2006). Changing patterns of sexual behaviour in the era of highly active antiretroviral therapy. *Current Opinion in Infectious Diseases*.

[3] Jin F., Crawford J., Prestage G. P. (2009). Unprotected anal intercourse, risk reduction behaviours, and subsequent HIV infection in a cohort of homosexual men. *AIDS*.

[4] Snowden J. M., Raymond H. F., McFarland W. (2009). Prevalence of seroadaptive behaviours of men who have sex with men, San Francisco, 2004. *Sexually Transmitted Infections*.

[5] Wilson D. P., Regan D. G., Heymer K. J., Jin F., Prestage G. P., Grulich A. E. (2010). Serosorting may increase the risk of HIV acquisition among men who have sex with men. *Sexually Transmitted Diseases*.

[6] Butler D. M., Smith D. M. (2007). Serosorting can potentially increase HIV transmissions. *AIDS*.

[7] Golden M. R., Stekler J., Hughes J. P., Wood R. W. (2008). HIV serosorting in men who have sex with men: is it safe. *Journal of Acquired Immune Deficiency Syndromes*.

[8] Marks G., Millett G. A., Bingham T., Lauby J., Murrill C. S., Stueve A. (2010). Prevalence and protective value of serosorting and strategic positioning among black and latino men who have sex with men. *Sexually Transmitted Diseases*.

[9] Philip S. S., Yu X., Donnell D., Vittinghoff E., Buchbinder S. (2010). Serosorting is associated with a decreased risk of HIV seroconversion in the EXPLORE study cohort. *PLoS ONE*.

[10] Poudel K. C., Poudel-Tandukar K., Yasuoka J., Jimba M. (2007). HIV superinfection: another reason to avoid serosorting practice. *The Lancet*.

[11] Siconolfi D. E., Moeller R. W. (2007). Serosorting. *BETA*.

[12] Truong H. H. M., Kellogg T., Klausner J. D. (2006). Increases in sexually transmitted infections and sexual risk behaviour without a concurrent increase in HIV incidence among men who have sex with men in San Francisco: a suggestion of HIV serosorting?. *Sexually Transmitted Infections*.

[13] World Health Organization (2011). *Prevention and Treatment of HIV and Other Sexually Transmitted Infections Among Men Who Have Sex with Men and Transgender People: Recommendations for a Public Health Approach*.

[14] Moher D., Liberati A., Tetzlaff J., Altman D. G., PRISMA Group (2009). Preferred reporting items for systematic reviews and meta-analyses. The PRISMA statement. *Annals of Internal Medicine*.

[15] Denison J. A., O'Reilly K. R., Schmid G. P., Kennedy C. E., Sweat M. D. (2008). HIV voluntary counseling and testing and behavioral risk reduction in developing countries: a meta-analysis, 1990–2005. *AIDS and Behavior*.

[16] Kennedy C., O'Reilly K., Medley A., Sweat M. (2007). The impact of HIV treatment on risk behaviour in developing countries: a systematic review. *AIDS Care*.

[17] Guyatt G. H., Oxman A. D., Vist G. (2011). GRADE guidelines: 4. Rating the quality of evidence—study limitations (risk of bias). *Journal of Clinical Epidemiology*.

[18] Cooper H., Hedges L. V. (1994). *The Handbook of Research Synthesis*.

[19] Higgins J. P. T., Green S. (2008). *Cochrane Handbook for Systematic Reviews of Interventions*.

[20] Jin F., Prestage G. P., Templeton D. J. (2012). The impact of HIV seroadaptive behaviors on sexually transmissible infections in HIV-negative homosexual men in Sydney, Australia. *Sexually Transmitted Diseases*.

